# Application of a score system to evaluate the risk of malnutrition in a multiple hospital setting

**DOI:** 10.1186/1824-7288-39-81

**Published:** 2013-12-27

**Authors:** Maria Immacolata Spagnuolo, Ilaria Liguoro, Fabrizia Chiatto, Daniela Mambretti, Alfredo Guarino

**Affiliations:** 1 Department of Translational Medical Science – Section of Pediatrics, University of Naples “Federico II”, Naples, Italy

**Keywords:** Pediatrics, Children, Hospital malnutrition, Screening tool, Chronic disease

## Abstract

**Background:**

An increased but unpredictable risk of malnutrition is associated with hospitalization, especially in children with chronic diseases. We investigated the applicability of Screening Tool for Risk of Impaired Nutritional Status and Growth (STRONGkids), an instrument proposed to estimate the risk of malnutrition in hospitalized children. We also evaluated the role of age and co-morbidities as risk for malnutrition.

**Methods:**

The STRONGkids consists of 4 items providing a score that classifies a patient in low, moderate, high risk for malnutrition. A prospective observational multi-centre study was performed in 12 Italian hospitals. Children 1–18 years consecutively admitted and otherwise unselected were enrolled. Their STRONGkids score was obtained and compared with the actual nutritional status expressed as BMI and Height for Age SD-score.

**Results:**

Of 144 children (75 males, mean age 6.5 ± 4.5 years), 52 (36%) had an underlying chronic disease. According to STRONGkids, 46 (32%) children were at low risk, 76 (53%) at moderate risk and 22 (15%) at high risk for malnutrition. The latter had significantly lower Height for Age values (mean SD value -1.07 ± 2.08; p = 0.008) and BMI values (mean SD-values -0.79 ± 2.09; p = 0.0021) in comparison to other groups. However, only 29 children were actually malnourished.

**Conclusions:**

The STRONGkids is easy to administer. It is highly sensitive but not specific. It may be used as a very preliminary screening tool to be integrated with other clinical data in order to reliably predict the risk of malnutrition.

## Italian abstract

Please see Additional file 1 for translation of the abstract into Italian language.

## Background

Malnutrition is associated with negative outcomes for inpatients, including increased risks of infections [1,2], increased muscle loss [3], impaired wound healing, longer hospital stay and increased morbidity and mortality [4-6]. Malnutrition may be responsible for delayed recovery and need for intensive nursing care, thus increasing the cost of hospitalization [7].

Data on acute and chronic malnutrition of children admitted to hospital are strictly dependent on the criteria used for its definition [8]. Malnutrition rates from 6 to 19% have been reported in European countries such as UK, France, Germany and the Netherlands, reaching 40% in Turkey [9-13]. A recent Italian study evaluated the incidence of hospital-acquired malnutrition in 496 children admitted for diagnostic procedures, minor infections, or other episodic illness, and reported that children with a BMI Z-score < -2 SD on admission showed a mean BMI decrease at the end of their hospital stay that was significantly higher than those with a better nutritional condition at admission [14].

Assessment of nutritional status is not easy in pediatric practice and there is no single parameter to define malnutrition. Assessment of patients’ actual nutritional status only identifies those who are already malnourished [15], while early identification of children at risk for malnutrition could promote timely nutritional interventions, preventing the short and long-term consequences of malnutrition. Routine screening for nutritional risk in children is hampered by the lack of validated nutritional assessment protocols and evaluation of weight gain and growth velocity remains the standard method [16]. Several screening tools have been proposed to assess the risk of malnutrition but their application is hampered by the limited data and their acceptance for broad use [17]. A screening tool for nutritional risk in children, called Screening Tool for Risk Of impaired Nutritional status and Growth (STRONGkids), was successfully applied in the Netherlands [18]. Subsequently the STRONGkids was tested in patients with Intestinal Bowel Diseases (IBD) but its reliability was unclear [19,20].

Aim of the present study was to investigate the efficacy of STRONGkids instrument in a population of children consecutively admitted to 12 Italian hospitals. Sensitivity, specificity and predictivity were assessed by comparing the scores of risk with the actual nutritional status of children. The scores of STRONGkids were also correlated with risk factors for malnutrition including age, the specific etiology for which the child was admitted to hospital and the association with underlying chronic diseases in order to examine their possible role.

## Materials and methods

A prospective observational multi-centre study was performed in 12 hospitals in Campania region, Italy, (including one University hospital), covering virtually 70% of entire the pediatric population living in the Region, during the months of October-November 2012. Italian children from 1 to 18 years of age admitted to hospital for any disease, hence unselected were enrolled. Patients in intensive care were excluded. Reasons for admission were classified as infectious, gastrointestinal, respiratory, genetic/metabolic, neurological, oncological, trauma, surgical, cardiac, and others.

The STRONGkids consists of 4 items (1- high risk underlying disease, 2- clinical assessment, 3- nutritional intake and presence of vomit or diarrhea, 4- recent weight loss) and children are classified in one out of three classes for malnutrition (low, moderate, high risk) according to a specific 5-points scale (low = 0, moderate = 1–3, high = 4–5). Each item is allocated a score of 1–2 points as follows:

– High risk disease (2 points): underlying illness with a risk of malnutrition or major surgery planned. The conditions that can lead to nutritional risk listed in the STRONGkids are: anorexia nervosa, congenital heart diseases, celiac disease, expected major surgery, dysmaturity/prematurity, bronchopulmonary dysplasia (maximum age 2 years), cystic fibrosis, digestive fistula, inflammatory bowel disease, infectious disease, metabolic disease, cancer, pancreatitis, chronic liver disease, muscle disease, chronic kidney disease, mental handicap/retardation, sepsis, short bowel syndrome, trauma, burns, other (specified by physician) [18].

– Subjective clinical assessment (1 point): poor nutritional status as judged by subjective clinical assessment (decreased subcutaneous fat and/or muscle mass and/or hollow face).

– Nutritional intake and losses (1 point): a) presence of diarrhoea with ≥ 5 stools/day and/or vomiting with >3 times/day in the last few days or b) reduced food intake in the last few days before admission or c) advised nutritional intervention or d) inability to receive adequate colonic intake because of pain.

– Weight loss or poor weight gain (1 point): weight loss or no weight gain (infants <1 year) in the last few weeks/months.

The questionnaires were administered by a nurse on one predetermined day, thus including all patients admitted that day, and collected by the study coordinating nurse (DM).

Anthropometric measurements were taken at admission and compared with published standard values obtained in an Italian reference pediatric population [21]. The STRONGkids score of each child was compared with his/her actual nutritional status expressed as BMI SD-score and Height-for-Age (HFA) SD-score. SD-scores < -2 for BMI and HFA were considered hallmarks of acute [22] and chronic malnutrition [23] respectively. Malnutrition rate was defined as the presence of acute and/or chronic malnutrition.

Informed consent was obtained from parents of enrolled children and the Ethics Committee of University of Naples ‘Federico II’ approved the protocol.

### Statistical analysis

Data were expressed as number/percent or as mean ± SD. Descriptive analyses were used to describe the study population and BMI was expressed as SD-score. Comparison of continuous data between groups was carried out using the *t*-test. The *χ*2 method, or the exact Fisher’s test when appropriate, was applied to compare the presence of chronic conditions and the reasons for admission. Sensitivity, specificity, Positive Predictive Value (PPV) and Negative Predictive Value (NPV) of STRONGkids were calculated based on BMI and HFA SD-scores, using their cut-off values. Univariate regression analysis was applied to identify the main factors associated with the following outcomes of interest: acute, chronic and overall malnutrition and high risk class of malnutrition according to STRONGkids. P (two-sided) <0.05 was considered as significant. Data were analyzed with the SPSS package version 17.0.

## Results

### General features

**Figure 1:**
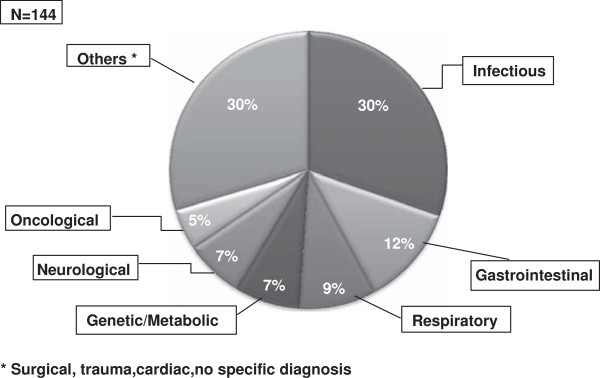
**Etiology distribution of children according to hospital setting.** The most common cause of admissions was an acute infectious disease. However, chronic gastrointestinal conditions, including Inflammatory Bowel Disease, and respiratory conditions were as common. Most heterogeneous conditions labelled as ‘Others’ were acute conditions. Therefore, overall chronic and acute conditions were equally represented in the observed population.

A total of 144 children (75 males mean age 6.5 ± 4.5 years) were enrolled (Table 1). Sixty patients were admitted in a University Children’s hospital and their data were recorded in 4 different days, whereas the other 84 were enrolled in 11 general hospitals in a single day of observation. Overall 52/144 (36%) of the hospitalized children suffered from an underlying chronic disease (Table 1). One third of children were admitted for an infectious disease (43/144). Non-infectious gastrointestinal (such as IBD) or respiratory (such as asthma) conditions were other common etiologies of admission (Figure 1).

**Table 1:** General features of children

**Patient characteristics**	
Sex, M:F (%)	52:48
Mean age (years) ± SD (95% CI)	6,5±4,5 (5.7–7.2)
Hospital, University: General (%)	42:58
Mean SD-scores BMI ±SD (95% CI)	0.05±1.85 (-0.2–0.3)
Mean SD-scores HFA ±SD (95% CI)	-0.37±1.84 (-0.68–0.06)
Underlying diseases n/N (%)	52/144 (36)

### STRONGkids scores and anthropometrics

According to STRONGkids score, 46 (32%) children were at low risk, 76 (53%) at moderate risk and 22 (15%) at high risk to develop malnutrition. Twenty-nine (20%) children were malnourished according to BMI (16/144; 11%) and HFA SD-scores (15/144; 10%), including 2 patients who met the criteria for both acute and chronic malnutrition (Table 2). However, only 5 of these 29 malnourished children (17%) were classified at high risk by STRONGkids. No difference was found in the incidence of acute and chronic malnutrition between risk classes. Mean SD-scores for HFA and BMI of the 144 children were -0.37 ± 1.85 and 0.05 ± 1.86 respectively (Table 3). Children with high risk STRONGkids score had significantly lower SD-scores for BMI (-0.79 ± 2.09; p =0.002) and for HFA (-1.07 ± 2.08; p = 0.008) in comparison with other groups (Table 3).

**Table 2:** Distribution of children with acute and chronic malnutrition in the STRONGkids classes of risk for malnutrition

	**Low**	**Moderate**	**High**	**Tot**
	**(N=46)**	**(N=76)**	**(N=22)**	**(N=144)**
Acute malnutrition (BMI SD-scores < -2)	5 (11%)	9 (12%)	2 (9%)	16 (11%)
Chronic malnutrition (HFA SD-scores < -2)	2 (4%)	9 (12%)	4 (18%)	15 (10%)
Overall malnutrition (Acute+Chronic)	7 (15%)	17^a^ (22%)	5^a^ (22%)	29^a^ (20%)

^a^two patients were classified with both acute and chronic malnutrition.

**Table 3:** STRONGkids classes of risk for malnutrition and mean anthropometric measurements

	**Low**	**Moderate**	**High**	**Tot**
	**(N=46)**	**(N=76)**	**(N=22)**	**(N=144)**
Mean SD-scores BMI ± SD	0.50±1.90	0.02±1.66	-0.79±2.09^a^	0.05±1.86
Mean SD-scores HFA ± SD	0.26±1.19	-0.58±2.01	-1.07±2.08^b^	-0.37±1.85
Sensitivity (95% CI)	34% (25–43)	71% (48–89)	86% (78–92)	71% (48–89)
Specificity (95% CI)	75% (55–89)	53% (43–63)	21% (8–41)	53% (43–63)
Positive predictive value (95% CI)	9% (5–13)	21% (17–25)	28% (19–37)	21% (17–25)
Negative predictive value (95% CI)	73% (69–77)	85% (85–90)	82% (79–85)	85% (85–90)

^a^Significantly lower SD-BMI compared to low and moderate categories (p = .0021).

^b^Significantly lower SD-HFA compared to low and moderate categories (p = .008).

**Figure 2:**
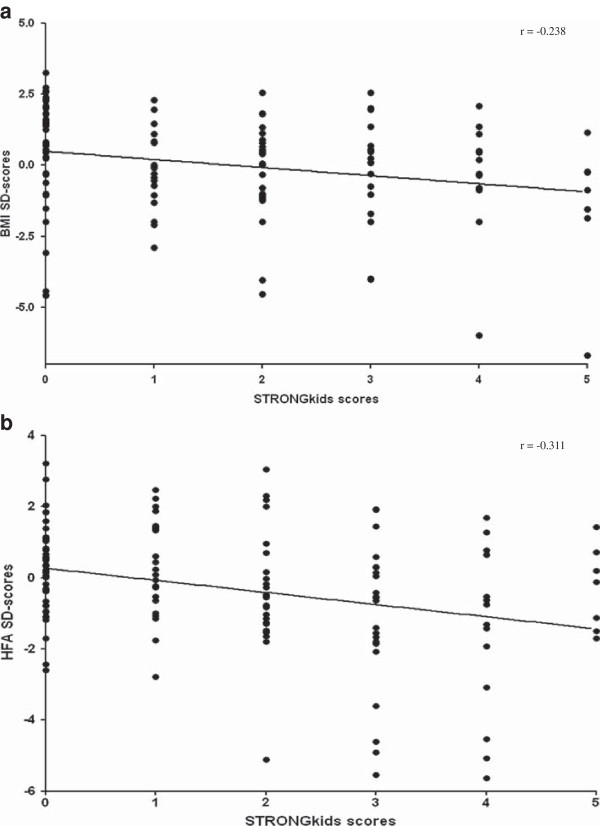
**Linear correlation between anthropometric measurements and STRONGkids scores. (a)** Correlation with BMI SD-scores. **(b)** Correlation with Height-for-Age SD-scores.

A weak albeit significant linear correlation was found between the STRONGkids scores and anthropometric measurements, considering both BMI SD-scores (r = -0.238; p = 0.0065) and HFA SD-scores (r = -0.311; p = 0.0002) (Figure 2a-b).

### Sensitivity, specificity and predictive values

Scores in the range of medium plus high risk (1–5) identified children at risk for malnutrition with a 71% sensitivity (95% CI: 48–89) and 53% specificity (95% CI: 43–63) (LR 1.5; p = 0.032) based on anthropometrics. The positive predictive value of the same scores was 21% (95% CI: 17–25) and their negative predictive value was 85% (95% CI: 85–90). Sensitivity, specificity and predictive values of the STRONGkids classes are provided in Table 3.

### Risk factors for malnutrition according to anthropometrics

The role of putative risk factors for malnutrition was also evaluated by analyzing the distribution of selected variables (Table 4). Children ≤ 5 years of age had a significantly higher risk to develop malnutrition (OR = 2.708; p = 0.024), particularly acute malnutrition (OR = 4.602; p = 0.006). The presence of underlying diseases also contributed to the development of malnutrition (OR = 10.234; p = 0.036), and overall the etiology played a role: children with a diagnosis of genetic disease had a very significantly higher risk to develop chronic malnutrition (OR = 10.167; p = 0.002) than other diagnostic groups. Furthermore, children with a diagnosis of gastrointestinal diseases (such as IBD) were more frequently classified at high nutritional risk by STRONGkids (OR = 3.75; p = 0.026) (Table 4).

**Table 4:** Risk factors for malnutrition

**Determinant**	**Acute malnutrition**		**Chronic malnutrition**		**Overall malnutrition**		**High risk class**	
	**(BMI SD-score <-2)**		**HFA SD-score <-2**					
	***OR***	***p***	***OR***	***p***	***OR***	***p***	***OR***	***p***
	***(95% CI)***		***(95% CI)***		***(95% CI)***		***(95% CI)***	
Age ≤ 5 years	4.602 (1.43–14.77)	.006^a^	1.295 (0.44–3.78)	.42	2.708 (1.16–6.31)	.024^b^	1.393 (0.56–3.47)	.496
Any underlying disease	1.841 (0.68–4.97)	.297	2.18 (0.72–6.19)	.257	1.04 (1.01-2)	.036^c^	10.234 (3.76–28.91)	.001
Genetic disease	2.925 (0.7–12.24)	.145	10.167 (2.63–39.24)	.002^d^	8.293 (2.24–30.68)	.002^e^	0.529 (0.06–4.35)	.547
Gastrointestinal disease	1.01 (0.3–1.59)	.129	1.89 (0.7–5.23)	.216	1.55 (1.22–3.15)	.023^f^	3.75 (1.22–11.5)	.026^g^

^a-b-c-d-e-f-g^p <0.05.

## Discussion

A number of screening tools have been proposed and their features were recently reviewed [17]. Reliability (e.g. predictivity) and acceptance by both health care workers and patients are major factors for success of a specific tool. However, in our population of approximately 150 children in 12 different hospitals, a total of 70% were at moderate or high nutritional risk according to STRONGkids, but only approximately 20% were actually malnourished according to anthropometric measurements. Even if the STRONGkids has been designed to assess the risk and not the actual presence of malnutrition, the gap between the actual incidence and the estimated risk was substantial. It should be considered that the STRONGkids score assigns 2 points (out of the maximum total of 5) to a patient with a reported underlying illness, which is by itself sufficient to include him/her in the moderate class for risk. Although the presence of chronic disease is associated with high risk for complications during hospitalization [24,25], including malnutrition [26], when we looked at their distribution in the STRONGkids, several conditions with a putative intrinsic nutritional risk were not associated with malnutrition. For example, celiac disease (when adequately controlled) [27] or mental retardation, do not necessarily imply a nutritional risk, while congenital heart diseases is usually resolved within the first months of life, but its presence in the patient clinical history is sufficient to put the child in the high risk class for malnutrition.

We found a significant but weak correlation between the STRONGkids score and the parameters of acute and chronic malnutrition. According to our results, the correlation between the STRONGkids and HFA-SD scores (hallmark of chronic malnutrition) was slightly stronger than that between the STRONGkids and BMI-SD scores (index of acute malnutrition). This appears to be in contrast with another study [28] in which the STRONGkids was significantly related with both BMI and HFA. In the latter study the STRONGkids provided more reliable information compared to the STAMP (Screening Tool for the Assessment of Malnutrition in Pediatrics) [29]. A possible explanation is the different weight of the item “underlying disease” in the two scoring systems. The STRONGkids includes a large list of chronic conditions, while in the STAMP there is the generic question “Does the child have a diagnosis that has any nutritional implications?”

Significant correlations between anthropometric measurements and the STRONGkids’ scores were found only for the high risk group, thus confirming that only children at high nutritional risk were actually already malnourished. Although less reliable compared to what reported in the Dutch study, the STRONGkids showed a high negative predictive value. In contrast the positive predictive value was low.

When we examined the risk factors associated with the risk of malnutrition, an age ≤ 5 years was associated with a higher risk of acute malnutrition, probably because younger children have a higher incidence of acute hospitalization-related malnutrition [30,31].

Specific etiologies were associated with a risk of malnutrition, including genetic/metabolic diseases. Also children with gastrointestinal diseases (especially those with IBD) were more likely to have scores in high risk class. A recent study in 46 patients with IBD showed the limits of several nutritional screening tools and pointed out that children with IBD are at high nutritional risk: many are underweight, even though the majority is of normal weight and some are overweight for their height [20]. We found similar results in our study.

We believe that the STRONGkids scores should be considered together with clinical and anthropometric data. In fact, many pediatricians who were involved in this study reported that the major limit of the instrument was the mismatch between their clinical judgement of patient’s actual nutritional risk and the categorization (high, moderate or low) deriving from the STRONGkids assessment (data not shown).

Tools, such as the Subjective Global nutritional Assessment (SGA) proposed by Secker and Jeejeebhoy [32,33], include a specific evaluation of physical parameters, objectively looking at signs of fat and muscle wasting (i.e. edema). In contrast, the STRONGkids does not include an objective assessment and it should be regarded as a very preliminary screening to be collected in the history and integrated with other clinical data in order to reliably predict the risk of malnutrition.

Routine screening for nutritional risk in children is currently hampered by a lack of validated and easy methods for nutritional assessment. In addition to the STRONGkids, other tools were designed in order to evaluate nutritional risk, but each of them showed some limits. The “Simple Pediatric Nutritional Risk Score” [34] (SPNRS) and the SGA tools [32,33] are considered too complicated and time-consuming and consequently their uptake has been limited [17]. Gerasimidis et al. [35] developed the Paediatric Yorkhill Malnutrition Score (PYMS), which is a four-stage evaluation, considering the BMI value, recent weight loss, decreased intake in the previous week, and expected affected nutrition by the admission/condition for the next week. Nutrition screening by nurses using the new PYMS score is feasible for pediatric inpatients, identifies children at risk of malnutrition and efficiently uses available resources.

However, in a recent study the STRONGkids was compared to PYMS and STAMP and it was the only tool that recognized all undernourished children in its medium or high risk groups [36].

In conclusion, the main positive feature of STRONGkids consists in its simple structure which makes it easy to use in any hospital setting. However its reliability and efficacy are limited. STRONGkids effectively drives attention towards important issues related to nutritional risk. Probably the major limit is that the score appears affected by the high scores given to underlying diseases, that however are associated with a true nutritional risk only when they are “active”. These limits might be improved with some modifications in the classification of patients, in order to identify children at actual nutritional risk. The main modification should be re-evaluation of the score given to chronic condition.

## Abbreviations

ART: Antiretroviral therapy; CI: Confidence interval for data estimates; Crl: Credibility interval for model projections – defined as the 2.5 to 97.5% percentile range; DALY: Disability adjusted life year; DfID: Department for International Development; FSW: Female sex workers; HIV: Human immunodeficiency virus; HSV-2: Herpes simplex type 2; ICER: Incremental cost-effectiveness ratio; IPM: International Partnership for Microbicides; MDP: Microbicide development programme.

## Competing interests

The authors declare that they have no competing interests.

## Authors’ contribution

MIS and AG conceived of and designed the study. DM collected all the data as coordinating nurse. MIS, IL and FC analyzed the data. MIS wrote the first draft of the manuscript. AG, IL and FC contributed to the writing of the manuscript. MIS, IL, FC, DM and AG agreed with the manuscript results. All authors read and approved the final manuscript.

## Supplementary Material

Additional file 1 **Italian abstract**. Click here for file
