# Supplementary material for: Application of a score system to evaluate the risk of malnutrition in a multiple hospital setting
Source: Ital J Pediatr. 2013 Dec 27;39:81. doi: 10.1186/1824-7288-39-81 (PMC3901031; doi:10.1186/1824-7288-39-81)
Supplement: Additional file 1 — Italian abstract. [file 1824-7288-39-81-S1.docx]

**ABSTRACT – Italian version**

**Background.** Un rischio aumentato di malnutrizione ma difficile da predire, si associa con l’ospedalizzazione, soprattutto nei bambini con malattie croniche. Abbiamo studiato l’applicabilità dello “Screening Tool for Risk Of Impaired Nutritional Status and Growth” (STRONGkids), uno strumento proposto con lo scopo di stimare il rischio di malnutrizione nei bambini ospedalizzati. Abbiamo anche valutato il ruolo dell’età e delle comorbidità come eventuali fattori di rischio per la malnutrizione**.**

**Metodi.** Lo strumento STRONGkids consiste di 4 elementi, ciascuno dei quali fornisce un punteggio che classifica il paziente a basso, moderato o alto rischio di malnutrizione. Uno studio osservazionale prospettico multicentrico è stato condotto in 12 ospedali italiani. Sono stati arruolati bambini di età compresa tra 1-18 anni ricoverati consecutivamente e pertanto non selezionati. Il punteggio ottenuto con STRONGkids per ogni paziente è stato confrontato con il loro reale stato nutrizionale espresso come BMI e altezza per età in z-score.

**Risultati.** Dei 144 bambini arruolati (75 maschi, età media 6.5±4.5 anni), 52 (36%) avevano una patologia cronica di base. Secondo STRONGkids, 46 (32%) bambini erano a basso rischio di malnutrizione, 76 (53%) a rischio moderato e 22 (15%) a rischio elevato. Questi ultimi avevano valori di altezza per età (z-score -1.07±2.08; p = 0.008) e BMI (z-score -0.79±2.09; p = 0.0021) significativamente più bassi rispetto agli altri gruppi. Tuttavia, solo 29 bambini sono risultati effettivamente malnutriti.

**Conclusioni.** STRONGkids è uno strumento facile da gestire. È altamente sensibile ma non specifico. Potrebbe essere utilizzato quale questionario preliminare da integrarsi con altri dati clinici allo scopo di predire in maniera affidabile il rischio di malnutrizione.
